# Accommodative responses stimulated from the Maddox components of vergence in participants with normal binocular vision

**DOI:** 10.1167/jov.25.8.3

**Published:** 2025-07-01

**Authors:** Sebastian N. Fine, Thomas Rutkowski, Elio M. Santos, Suril Gohel, Farzin Hajebrahimi, Mitchell Scheiman, Tara L. Alvarez

**Affiliations:** 1 Department of Biomedical Engineering, New Jersey Institute of Technology, Newark, NJ, USA; 2 Department of Health Informatics, Rutgers University School of Health Professions, Newark, NJ, USA; 3 Pennsylvania College of Optometry, Salus at Drexel University, Elkins Park, PA, USA

**Keywords:** accommodation, maddox components, normal binocular vision, proximal, vergence

## Abstract

Understanding the interplay of responses to stimulated accommodative blur (B), disparity (D), proximal (P), and diminished blur (-b), disparity (-d), and proximal (-p) cueing within binocularly normal participants is important for comparisons to patient populations. Recordings from 31 participants enrolled in the Convergence Insufficiency Neuro-mechanism Adult Population Study (NCT03593031) were collected. After artifact removal, analyses were performed on 20 BDP, 22 BD(-p), 27 BP(-d), 29 DP(-b), 24 B(-dp), 31 D(-bp), and 29 P(-bd) participant-level response datasets. Group-level statistics were assessed to evaluate the main effect of cue conditions on peak velocity (diopters/second) and final amplitude (diopters). Peak velocity assesses the preprogrammed portion of accommodation, whereas final amplitude assesses the feedback portion of accommodation. Post hoc pairwise comparisons were used to determine cue-to-cue significance. Significant main effects were found for final amplitude and peak velocity metrics (*p* < 0.05), indicating differences across cue conditions. Responses evoked by blur and disparity were comparable to those responses with all cues (BDP) for both far-to-near and near-to-far transitions. Responses evoked by blur or disparity cues elicited a reduced accommodative response, as indicated by peak velocity and final amplitude, compared to responses from blur and disparity cues. Blur and disparity cues can stimulate accommodative responses through the convergence accommodative/convergence crosslink. Results support significant contributions from blur and disparity cueing to accommodative responses compared with the proximal cue. This research forms the foundation for comparing accommodative responses in individuals with binocular vision dysfunctions.

## Introduction

In daily life, it is common for a person to quickly fixate from far to near, such as looking at a whiteboard in a classroom (far target) and then fixating on a computer or book (near target), or looking at the dashboard in their car (near target) and then to a road sign (far target). Daily visual tasks require coordination of the oculomotor and accommodative systems to establish a single (binocularly fused) and clear (focused onto the retina) image in the brain. Three visual cues provide the stimulation necessary to view objects as single and clear in natural viewing conditions. First, retinal disparity is stimulated by the differences in the viewing perspectives of each eye. The second is blur to drive modulation in the convexity of the biological crystalline lens geometry to focus the emitted light of a visual target onto the retina. Finally, there is visual proximity, where the size of the visual target appears to differ based on the perceived distance from the viewer. Collectively, disparity, blur, and proximal visual cues are known as the Maddox components of the vergence system (Maddox, 1893). The literature supports that the influence of disparity vergence on accommodation through the convergence accommodative/convergence (CA/C) crosslink is dependent on the velocity of the ramp stimuli (Schor & Kotulak, 1986). Yet, it is unclear whether the Maddox components of vergence stimulated during slow tracking (Horwood & Riddell, 2008) will stimulate the accommodative system in the same or different manner for quickly changing step stimuli.

Under natural viewing conditions, the vergence, accommodation, and pupil systems are simultaneously stimulated, forming the near triad. Yet, in the laboratory, these systems can be stimulated independently to study the influence that one system has on another. This investigation concentrated on the influence of the Maddox components on the accommodation system via quantitative accommodation responses. One of the first models describing the interaction between vergence and accommodation was proposed by Westheimer (1955). Numerous investigations have further developed the crosslink model of vergence and accommodation (Bharadwaj & Candy, 2009; Hung, 1991; Hung & Semmlow, 1980; Hung, Semmlow, & Ciuffreda, 1983; Krishnan, Phillips, & Stark, 1973; Krishnan & Stark, 1977; Maxwell, Tong, & Schor, 2010; Oh, Lee, & Bovik, 2016; Schor & Kotulak, 1986; Semmlow & Wetzel, 1979). Prior research has shown that these cues interact nonlinearly to drive accommodation (Cumming & Judge, 1986). Briefly, within the laboratory, when disparity is open looped, blur-driven accommodation initiates a vergence response, which is referred to as accommodative convergence (AC). Analogously, when blur is open looped, disparity-driven vergence initiates an accommodative response referred to as convergence accommodation (CA) (Bharadwaj & Candy, 2009; Held, Cooper, & Banks, 2012; Hung et al., 1983; Hung, Ciuffreda, Semmlow, & Horng, 1994; Ittelson & Ames, 1961; Kasthurirangan & Glasser, 2005; Schor, Alexander, Cormack, & Stevenson, 1992; Schor, Lott, Pope, & Graham, 1999). This investigation also stimulated proximal cues to study the impact proximal cues have on the accommodative system.

The accommodative system has been modeled using a combination of preprogrammed and feedback control (Maxwell et al., 2010; Oh et al., 2016; Schor & Bharadwaj, 2005; Schor & Bharadwaj, 2006) with many behavioral research studies to validate the model (Bharadwaj & Candy, 2009; Bharadwaj & Schor, 2005; Gamlin, 1999; Gamlin & Reiner, 1991; Held et al., 2012; Horwood & Riddell, 2008; Ittelson & Ames, 1961; Mays & Gamlin, 1995; Mays, Porter, Gamlin, & Tello, 1986; Ramsdale & Charman, 1988; Schor et al., 1992; Schor & Kotulak, 1986). Prior research has concentrated on the feedback-controlled portions of the vergence and accommodative systems (Horwood & Riddell, 2008) by studying smoothly tracking stimuli presented on midline. The novelty of this present study is to probe how the three Maddox cues, isolated and in combination, modify the preprogrammed portions of the accommodation system assessed via the peak velocity and the feedback portion assessed via the final amplitude of the accommodation response. This present research is synergistic with prior studies (Horwood & Riddell, 2008) by assessing the final amplitude of responses, but is also unique in that it concentrated on the preprogrammed portions of the accommodative responses assessed via peak velocity. Horwood and Riddell demonstrated that, for the feedback-controlled portion of accommodation, blur and disparity are the dominant cues compared to the proximal cue assessed by final amplitude. This study aimed to test the hypothesis that, for the preprogrammed portion of accommodative responses, as assessed by peak velocity from step-response stimuli, transitioning from visually near to far or from far to near the primary cues will be blur and disparity, and the secondary cue will be proximal.

## Methods

### Study design

This investigation is a part of the Convergence Insufficiency Neuro-mechanism Adult Population Study (CINAPS) randomized clinical trial registered on ClinicalTrials.gov (NCT03593031). Previous publications have discussed the study design for CINAPS (Alvarez et al., 2020). The Institutional Review Board of the New Jersey Institute of Technology approved the study protocol (HHS FWA 00003246) in accordance with the tenets of the Declaration of Helsinki. Before enrolling, all participants provided written informed consent.

### Participant eligibility

All definitions complied with the CINAPS study design and are described previously (Alvarez et al., 2020). To briefly summarize, binocularly normal visual participants between 18 and 35 years of age were defined as having the following: (a) 20/25, or better, corrected visual acuity, (b) Convergence Insufficiency Symptom Survey (CISS) (Convergence Insufficiency Treatment Trial [CITT] Study Group, 2008) score of less than 21 points, (c) normal amplitude of accommodation based on Hofstetter's formula (Hofstetter, 1950), (d) random-dot stereopsis global level of 500 seconds of arc (arcsec) or better and local stereopsis of 70 arcsec or better, (e) near point of convergence less than 6 cm measured from the bridge of the nose along midline, and (f) normal positive fusional vergence (PFV) based on Sheard's criterion (PFV at least twice the near dissociated phoria).

### Data acquisition

**Figure 1.**
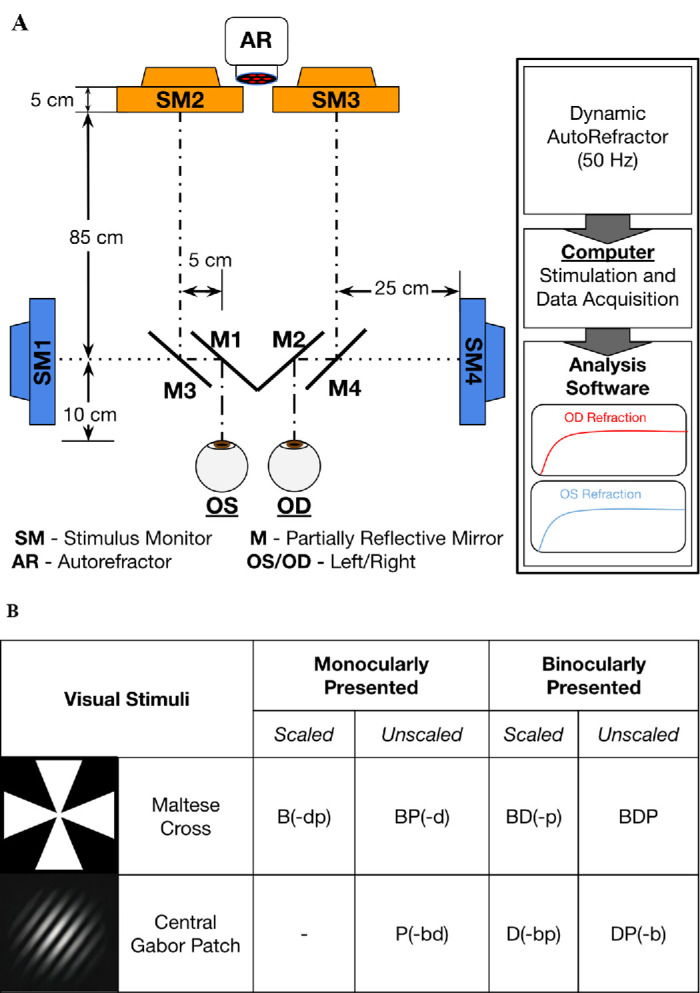
(**A**) Experimental recording configuration of the haploscope with four semi-reflective mirrors (M1 to M4) that enables selectively presenting monocular stimuli via two stimuli monitors (SM3 and SM4) for the right eye (OD) or via two stimuli monitors (SM1 and SM2) for the left eye (OS), at near (40 cm) and at far (1 meter). (**B**) The presentation type and visual stimuli of each viewing condition.

A PowerRef 3 (Plusoptix, Nuremberg, Germany) dynamic autorefractor sampling at 50 Hz was utilized for this study. The autorefractor was set at an optical distance of 1 meter from the participant per the manufacturer's configuration recommendations with partially reflective mirrors to view presented stimuli, as seen in Figure 1a. Participants were situated within a haploscope to enable independent presentation of visual stimuli to one or both eyes, with all stimuli presented along the midline. Binocular recordings of each eye were captured regardless of whether the visual stimuli were monocularly or binocularly presented. The Plusoptix software required manual triggering by the operator for the onset and conclusion of each cue recording session. For each eye movement recording, unique consideration of the stimuli characteristics to stimulate or diminish combinations of the Maddox cues was given (Figure 1b). For each stimulated movement and cue condition, 15 binocular far to near and 15 near to far were interleaved, with 30 total movements recorded for each of the following cues: D(-bp), DB(-p), DP(-b), and BDP. Monocularly, 15 far to near and 15 near to farwere interleaved per eye, with 60 total accommodative movements recorded for each of the following cues: B(-dp), P(-bd), and BP(-d). Per-subject level averages were obtained for each cue following assessment for the validity of each individual recording (non-response, noise, or signal recording artifacts were omitted). Individual responses without artifacts were subsequently utilized for group-level statistics.

Two primary stimuli were utilized within this experiment: a high-acuity Maltese Cross and a low-acuity (foveal) 2° target eccentricity Gabor patch (see Figure 1b). The construction of the Gabor patch to diminish accommodative response was based on prior literature (Schor & Kotulak, 1986) using the following formula: Stimulus(*x*) = 3 exp(–*x*^2^/σ^2^) – 2 exp(–*x*^2^/2.25σ^2^) + *K*, in which σ is the space constant and *K* equals the mean luminance. The Gabor patch used here is analogous to visual stimuli used by prior investigations (Horwood & Riddell, 2008). The selective presentation of these targets enabled stimulation of isolated or combinatory vergence and accommodative cue responses. A disparity stimulus (D) requires the binocular presentation of a visual target, whereas the monocular presentation of a stimulus diminishes this response (-d). A blur stimulus (B) utilizes a visually acute target, such as a Maltese Cross, and can be diminished (-b) using a low-acuity target, such as a Gabor patch. A proximal stimulus (P) can use a looming stimulus and can be diminished (-p) by the scaling of a stimulus based on its optical distance from the viewer to appear unchanged in perceived size. Prior literature supports that depth can be interpreted based on disparity or blur cues (Held et al., 2012). For this current study, proximal awareness was reduced using a darkened visual environment within the haploscope, and all lights from instrumentation were blocked, as this has been shown to diminish proximal awareness (Rosenfield & Ciuffreda, 1991).

A total of seven unique combinations of cues were stimulated per experimental recording sessions: B(-dp), P(-bd), D(-bp), BP(-d), BD(-p), DP(-b), and BDP. The accommodative change between the near stimulus monitors (SM1 or SM4) located at 40 cm, or 2.5 accommodative diopter demand, and the far stimulus monitors (SM2 or SM3) at 1 meter, or 1 accommodative diopter demand, induced an accommodative change of 1.5 diopters (D) for stimuli on the near stimulus monitor to the far stimulus monitor or vice versa. The change from far to near (1.5-D step change) stimulated the preprogramming portion of the accommodative system, followed by the feedback portion of the accommodative system. Presenting a scaled binocular Gabor patch with low spatial frequency stimulated the disparity cue while diminishing the accommodative and proximity information. Disparity-only cue D(-bp) and disparity with proximal DP(-b) were presented on the near monitors SM1 and SM4 in Figure 1a. The disparity stimulus at near (SM1 and SM4) or far (SM2 and SM3) has been shown to exhibit different responses that are dependent on the initial vergence angle (Alvarez, Bhavsar, Semmlow, Bergen, & Pedrono, 2005b; Patel, Jiang, White, & Ogmen, 1999). This study concentrated on the near disparity stimuli because future work is planned to study binocular dysfunctions in near visual space. To simulate a proximal-only cue, the Gabor patch was presented monocularly on the near monitor (SM1) for the left eye and then repeated on SM4 for the right eye, where it jumped in size. A summary of the visual stimuli (Maltese Cross or Gabor patch) for monocular or binocular viewing conditions and whether it is scaled or unscaled for each of the seven visual combinations is summarized in Figure 1a. A randomized delay in each stimulus presentation, between 500 ms and 2000 ms, was utilized to diminish anticipatory response effects from participants (Alvarez, Semmlow, Yuan, & Munoz, 2002; Alvarez, Semmlow, & Pedrono, 2005a; Yuan, Semmlow, & Munoz, 2000).

### Data processing

The Accommodative Movement Analysis Program (AMAP) (Fine et al., 2023) used standard outputs from the Plusoptix PowerRef 3 to evaluate signal recording integrity. Loss of signal within one eye was replaced by the accommodative response of the other eye if there was signal dropout. Experimentally, binocularly presented stimuli described above for far to near and near to far locations were recorded for 5 seconds per movement. File rejection criteria thresholds were conservatively set to the following: (a) data loss of 25%, or greater, per the total recorded sample points of a file, or (b) a file containing less than 15 seconds (750 data points) of recording where a minimum of 150 seconds (7500 data points) was expected. Files with 225 or greater consecutive missing samples (4.5 seconds) were omitted. A fifth-order low-pass Butterworth filter with a frequency cut-off of 20 Hz was applied to reduce noise from the powered electronics. The magnitude of alignment of all recorded data was determined by subtracting the refractive value or offset of the first recorded data point from each respective data point to plot the change in refraction.

Variability of the calibration slope has been reported to be dependent on race (Bharadwaj et al., 2013; Sravani, Nilagiri, & Bharadwaj, 2015). Calibration of the recorded accommodative movements per participant was attempted utilizing a series of lenses ranging from +8 D to –8 D in 1-D steps. However, recorded aberrations induced by the operator manually positioning each lens rendered participant-level calibrations non-viable, as shown by prior research (Thompson & Guyton, 1983; Thompson & Guyton, 1985). Hence, the internal calibration factor of the Plusoptix PowerRef 3 was adjusted by race per the calibration (Bharadwaj et al., 2013; Sravani et al., 2015).

**Figure 2.**
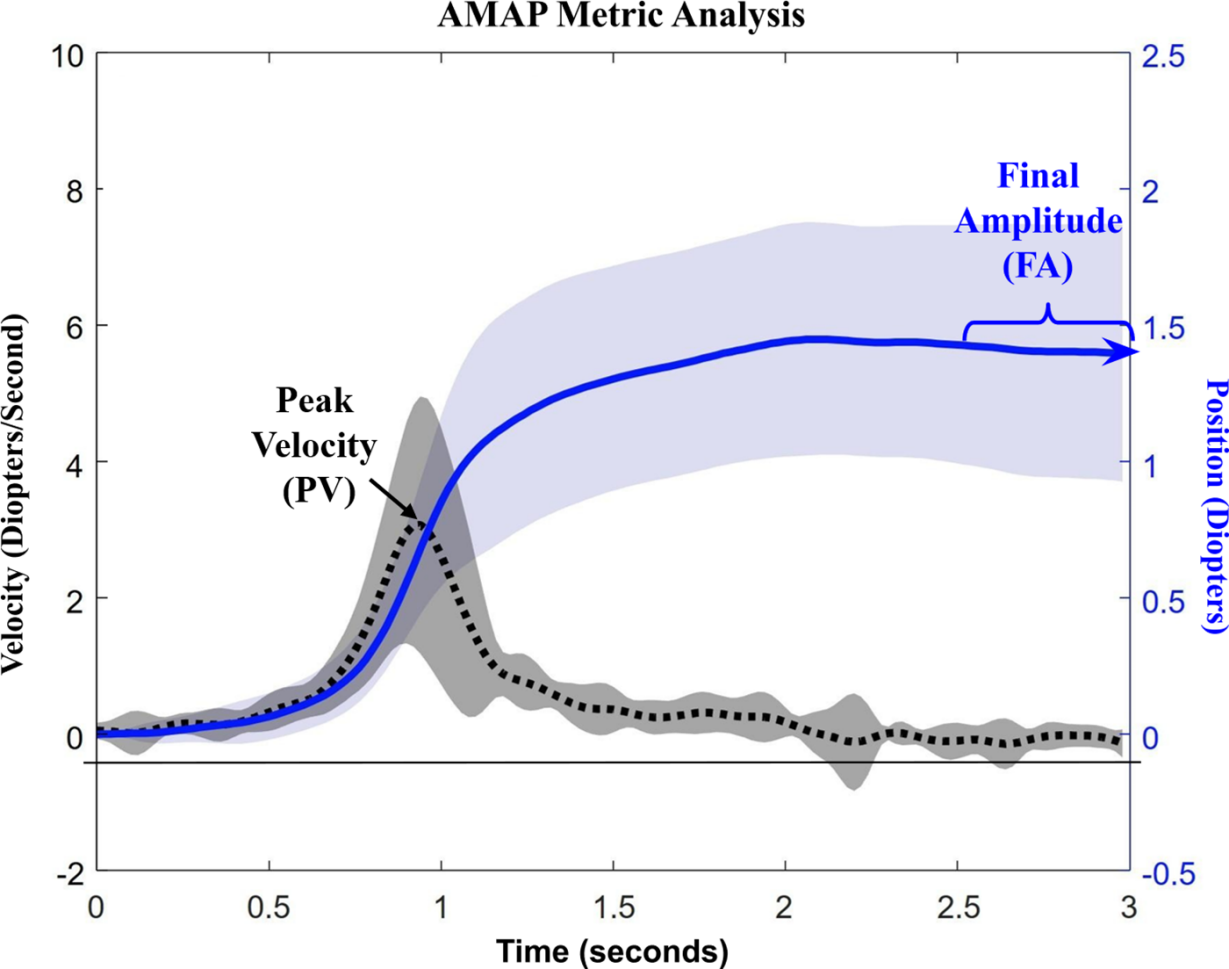
Visualization of the positional (solid blue line) and velocity (dashed black line) metrics assessed for the average recorded accommodative movement from a single participant. PV is the maximum velocity denoted by the black arrow. The FA is the average of the last half-second of the recording (25 samples). The shaded area is 1 *SD* for position (blue) and velocity (gray) responses for a typical single participant.

Figure 2 describes the key metrics evaluated for each accommodative response. Peak velocity (PV) denotes the maximum observed rate of change for each accommodative response. The final amplitude (FA) of the accommodative response was calculated as the average of the final 25 samples (half a second) of the recorded position. Metrics analyzed within AMAP were validated by data analysts for each eye position and the average response. Figure 2 is an example of a single participant and does not represent the group-level results, which differed from the typical participant presented.

### Statistical methods for group-level analysis

The jamovi software suite (https://www.jamovi.org) and a companion statistical software module (Fox et al., 2023) were utilized for statistical analysis to calculate the main effects of cue conditions and assess the statistical test assumptions. Levene's test (Levene, 1960) of homogeneous variance and Shapiro–Wilk's test (Shapiro & Wilk, 1965) were used to check for normality. Preliminary descriptive statistics for the resultant datasets revealed failures in equal variance (*p* < 0.05), which did not meet the assumptions for performing parametric one-way analysis of variance (ANOVA) to assess the main effect. Hence, to test for main effects and subsequent post hoc analyses, a non-parametric test was utilized to evaluate significance (Nahm, 2016). The Kruskal–Wallis, non-parametric, one-way ANOVA (Kruskal & Wallis, 1952) was selected to assess the potential main effect for the dependent variables: peak velocity and final amplitude. Upon observing a significant main effect, Dwass–Steel–Critchlow–Fligner (DSCF) post hoc pairwise comparisons (Douglas & Michael, 1991) were performed to assess the significance of differences between isolated and combined cues for the dependent variables: peak velocity and final amplitude. The utilization of the DSCF as a non-parametric post hoc test further provided specific advantages, as pairwise comparisons are integrated within the test corrected for multiple comparisons (Dunn, 1961; Dunn, 1964). Intra-participant level outliers, per cue stimulus, were identified as any accommodative movement that was 2 *SD* or more above or below a participant's movement mean and were removed from further analysis. Inter-participant level outliers were identified and labeled using 1.5 times the interquartile range (IQR) and were subsequently removed from comparisons. Utilizing the previously stated criteria, five outliers within the calibrated final amplitude—three BP(-d) and two P(-bd)—and five response outliers for peak velocity—one BDP, two BD(-p), and two BP(-d)—within focus were removed from further analysis. Defocus response outliers identified three for peak velocity, one BDP and two BD(-p); two for final amplitude, BP(-d), were also removed.

## Results

### Clinical demographics of participants

Appendix A provides tabularized clinically recorded metrics obtained through a comprehensive sensory-motor optometric exam performed by an optometrist (MS) to verify the binocular normalcy of enrolled participants. The participants, on average, were 21.7 years of age, with a standard deviation of 3.61 years (24 males and seven females). The self-reported ethnicity of the participants was as follows: 47% Asian (*n* = 15), 36% Caucasian (*n* = 11), 6% Black (*n* = 2), and 11% of the participants (*n* = 4) preferred not to answer, based on the reporting standards of the National Institutes of Health.

### Positional and velocity results

**Figure 3.**
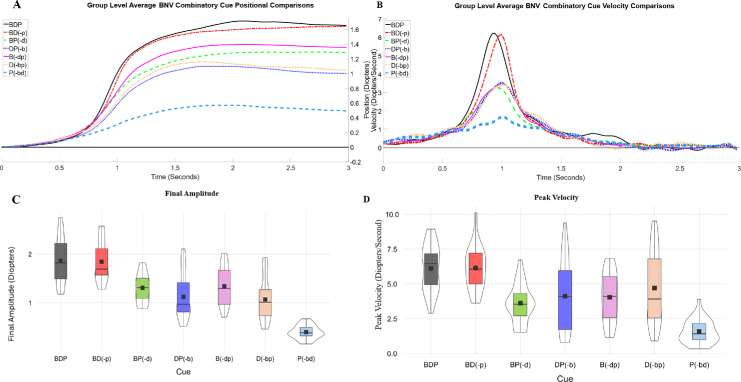
Group-level average responses per viewing condition aligned using accommodative peak velocity. (**A**, **B**) Accommodative movement positional (**A**) and velocity (**B**) averages for binocularly normal participants for each experimentally stimulated visual cue: BDP, black line trace; BD(-p), red dotted dashed trace; BP(-d), green dashed trace; DP(-b), dark blue dotted trace; B(-dp), pink line trace; D(-bp), peach dotted dashed trace; and P(-bd), light blue dashed trace. Descriptive violin plots demonstrate the numerical comparisons of final amplitude (**C**) and peak velocity (**D**) metrics for each experimentally stimulated visual cue.

A group-level average of the accommodative positional and velocity responses to the different stimulus cues for all participants is shown in Figure 3. Positional and velocity responses in Figures 3A and 3B provide visual differences in response magnitude for each analyzed cue combination. Violin plots of the per accommodative response analysis pooled for all participants with box and whisker plots are provided for the final amplitude (Figures 3C) and peak velocity (Figures 3D) with the median (solid black line) and the mean (black square) and interquartile distribution (box). Figure 3 shows that the averaged all-cues (BDP) group-level response (solid black line) and the combined disparity and blur cues, BD(-p) (red dashed-dotted line), group-level response were closest to attaining the stimulated target of 1.5 D for the final amplitude. Next, the averaged group-level responses that contained a disparity or blur cue reached between 1 D and 1.35 D, specifically the BP(-d) (green dashed line), DP(-b) (dark blue dotted line), B(-dp) (pink solid line), and D(-bp) (yellow dash-dotted line). Only when the isolated proximal cue P(-bd) (light blue dashed line) was stimulated was the final amplitude substantially below the stimulus target, reaching about 0.5 D.

Descriptive statistics for each metric are provided in Appendix B. The per-cue data failed the assumption of normality, as indicated by the Shapiro–Wilk test (*p* < 0.05), and equal variance for comparisons, as determined by the Levene test (*p* < 0.05). Hence, a Kruskal–Wallis non-parametric one-way ANOVA was performed. Subsequent post hoc comparisons evaluated the differences within each of the visual cues for focus accommodative responses and then repeated for defocus accommodative responses. The main effect assessing accommodative focus PV was significant, χ^2^(6) = 70, *p* < 0.0001, as was the FA, χ^2^(6) = 101, *p* < 0.0001. Similarly significant main effects were observed for accommodative defocus PV, χ^2^(6) = 86, *p* < 0.0001, and FA, χ^2^(6) = 107, *p* < 0.0001). Post hoc comparisons for each visual cue comparison for focus and defocus for PV and FA are summarized in Table 1.

**Table 1.** Summary of post hoc *p*-value results from DSCF tests for PV and FA for each cue and cue combination. D, defocus; F, focus.

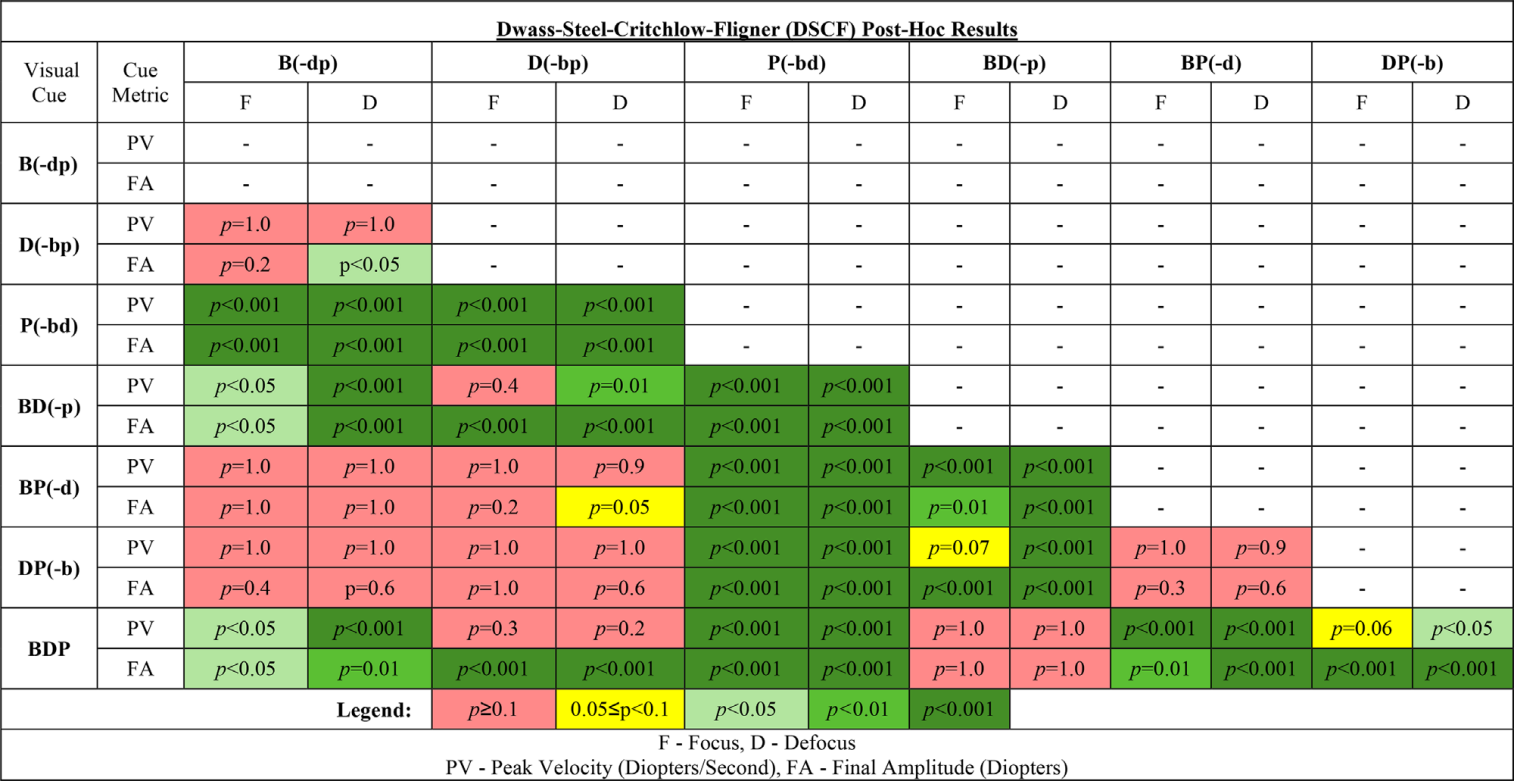

## Discussion

### Comparison to prior literature

The final amplitude and peak velocity metrics stimulated by isolated, paired, and combinatory visual cues of blur, disparity, and proximal stimuli (the Maddox components) provide different contributions to the accommodative response. Aligned with prior studies (Horwood & Riddell, 2008; Maxwell, Tong, & Schor, 2012), vergence and accommodation responses were observed in all conditions where disparity was present (Maxwell et al., 2010; Schor & Bharadwaj, 2005; Schor & Bharadwaj, 2006; Semmlow & Venkiteswaran, 1976; Semmlow & Wetzel, 1979; Wilson, 1973). The accommodative response without a direct blur stimulus reinforces prior findings of the dominant contribution of the disparity cue (Bharadwaj & Candy, 2009; Fincham & Walton, 1957; Horwood & Riddell, 2008; Ravisankar, Tyler, Schor, & Bharadwaj, 2024).

The final amplitude, as a metric, provides a method for evaluating how closely the response of the accommodative system response aligns with the experimentally stimulated cue demand and assesses the feedback component of the accommodative responses. Prior results report the final amplitude as a gain defined as the final recorded accommodative amplitude divided by the experimentally stimulated demand. A gain of 1.0 would represent a perfect response to the stimulus (Horwood & Riddell, 2013). Hence, if a 1.5-D stimulus is presented, then an ideal response would be a final amplitude of 1.5 D and a calculated gain of 1.0. Participants presented blur and disparity cues, BD(-p), had gain values close to 1.0. The addition of the proximal cue for the all cue responses BDP slightly reduced the gain values, but these differences were insignificant compared to the blur and disparity, BD(-p), response behaviors. However, for paired cues that contained disparity or blur, final amplitude gains were reduced to between 0.8 BP(-d) and 0.6 DP(-b), which share similar gain values with those of blur-only (0.71) and disparity-only (0.55) cues, respectively. Henceforth, these observations complement the previous literature, which has tracked developmental differences in the utilization and balance of disparity, blur, and proximal cues (Horwood & Riddell, 2013). The removal of the proximal cue may explain why the BD(-p) responses exhibited more variability in the final amplitude compared to the all cue condition of BDP. Yet overall, the proximal cue did not substantially contribute to the refractive response. In addition to the gain assessment, our results can be compared to the literature for the CA/C ratio through objective measures obtained using the Plusoptix disparity isolated cue, D(-bp), response final amplitude (in diopters) divided by the gaze final amplitude (in prism diopters, or ΔD). For our results, the CA/C ratio was 0.33 ± 0.10 D/ΔD for this study, which is similar to prior results of 0.2 D/ΔD (Horwood & Riddell, 2013).

Comparisons of combinatory and isolated cue conditions describe the significance of both blur and disparity cues for accommodative response peak velocity and final position. The isolated stimulation of blur, disparity, or proximal cues contributed significantly less to the final accommodation amplitude and peak velocity compared to the combined stimulation of blur and disparity cues. The insignificant difference in paired blur and proximal, as well as disparity and proximal, final amplitude metrics provides insight into the limited contributions of the proximal cue on response initiation but potentially aids in the slower sustaining component of final accommodative amplitude. Disparity-only responses demonstrate significant differences in the final accommodative amplitude compared with combinations of blur-only and blur with proximal responses. However, when disparity and blur are paired, the contributions of the proximal cue appear to be diminished, as no significant differences were observed between BD(-p) and BDP responses. These observations suggest that direct stimulation of the accommodative system elicits a more pronounced final sustained response than induced accommodative contributions from the CA/C crosslink. Demonstrated in paired cues and all cue viewing conditions, the removal of either blur (-b) or disparity (-d) cues showed a significant decrease in performance compared with all cue viewing conditions. These results support that the CA/C crosslink stimulates an accommodative response (Kim, Vicci, Granger-Donetti, & Alvarez, 2011; Schor & Kotulak, 1986) and that disparity, when present in isolation, can drive accommodation (Horwood & Riddell, 2008).

The novelty of this study lies in the assessment of the preprogrammed portion of the accommodative system using peak velocity. Similar to the feedback-controlled portion of the accommodative system, when blur and disparity are presented, the peak velocity of the responses is nearly the same as when all cues are present. Yet, when blur or disparity is present, a slower response is initiated, which proposes a smaller pulse height and width of the preprogrammed portion of the accommodative system. However, when only the proximal cue is simulated, the accommodative response is very slow, suggesting very little preprogrammed stimulation.

### Applications for clinical comparisons

A common binocular vision dysfunction known as convergence insufficiency (CI) hinders a patient's binocular coordination at near (Cooper & Jamal, 2012; Gantz & Stiebel-Kalish, 2022; Rouse et al., 1999). Further, CI has notable similarities in visual symptoms to accommodation insufficiency, such as blurred and haloing vision (Borsting et al., 2003; Hussaindeen & Murali, 2020). Between 4.2% and 17.6% of the general population are diagnosed with CI (Davis et al., 2016; Hussaindeen et al., 2017; Rouse et al., 1999; Wajuihian & Hansraj, 2016). A substantial subgroup of diagnosed CI patients are codiagnosed with accommodation insufficiency that varies based on age from approximately 3% in children (Nunes, Monteiro, Ferreira, & Nunes, 2019) to nearly 20% of young adults (Saeed et al., 2020). Diagnosis rates of binocular oculomotor and/or accommodative dysfunctions following mild traumatic brain injury with persistent post-concussion symptoms reach above 50% (Master et al., 2016). Furthermore, these oculomotor and accommodative pathways are affected by neurodegenerative diseases such as Parkinson's, Huntington's, and Alzheimer's, where binocular coordination and responses become degraded (Antoniades & Kennard, 2015; Garbutt et al., 2008). Understanding the interaction between vergence and accommodative mechanisms may have implications for therapeutic interventions.

The results of this study on binocularly normal accommodative response data in isolated, combinatory, and all cue viewing conditions enable establishing a comparison dataset for patients with binocular and/or accommodative vision dysfunctions. The importance of continuing to quantify binocularly normal vergence and accommodative function is seen in clinical settings, where primary eyecare optometrists interface with individuals who can express qualitative visual symptoms but require quantitative performance assessments for the clinician to make informed treatment decisions (Cooper et al., 2001). Hence, co-investigation of accommodative dysfunction and binocular vision disorders, such as convergence and accommodation insufficiency, excess, or infacility, can further our understanding of the dynamic interactions between vergence accommodation and accommodative vergence, as well as these systems in isolation.

Utilizing the insights gained from assessing accommodative responses in binocularly normal individuals, along with the established analytical techniques and tools, the implementation of these objective recording and analysis methods is feasible for use within a randomized clinical trial. Quantitative assessment of vergence and accommodative performance within the combinations of the Maddox cues across current standard therapeutic intervention methods, such as office-based vergence and accommodative therapy (Scheiman et al., 2015) or office-based accommodative with movement therapy (Alvarez et al., 2024), for individuals with binocular and accommodative dysfunction with or without concussion is warranted. The baseline and post-treatment outcomes can assess the mechanism of how the accommodative and vergence systems are remediated. Outcome measures from randomized clinical trials investigating therapeutic interventions for patients with vergence dysfunction or accommodative dysfunction, or both, can be compared with the results reported here to determine whether patients achieve similar performance to those with normal binocular vision. Future studies can determine whether patient compensatory mechanisms post-therapy are similar to individuals with binocularly normal vision or if different neural control strategies are developed post-treatment.

### Limitations and future directions

Future research will integrate the Plusoptix PowerRef3 software, an objective eye-tracking system, so that vergence eye movement responses can be synchronized with accommodative responses. Modifications to the instrumentation are underway to integrate a high temporal-resolution eye tracker with the Plusoptix 50-Hz sampling rate instrumentation, allowing for the simultaneous analysis of objective vergence eye movements and accommodative responses. There are limitations to controlling accommodation stimulation. Utilization of phenylephrine, or similar eye drops, administered in a controlled manner and the implications of dosage concentration (Esteve-Taboada et al., 2016) to control for pupillary response could be a future implementation. Future studies will also study the disparity cue at far (1-meter) presentations using a non-accommodative stimulus. Because the goal of this work was to establish a database of binocularly normal participants for comparison with patients with convergence insufficiency, our focus was on the near visual space. Furthermore, as detailed in previous literature (Horwood & Riddell, 2008), the spatial frequency of the Gabor patch utilized may be insufficient, and inherent limitations in resolution may not be sufficient to reduce visual cueing for an accommodative response. Although a no-target stimulation could present benefits, the inability to control participants’ attention toward a singular plane without a visual target may lead to inconsistent response behaviors and diminish the value it provides. The presentation of visual stimuli could also be randomized to reduce the influence of presentation order.

Future research should also include the measurement of the AC/A and CA/C ratios obtained clinically, allowing these measures to be compared with objective measurements. The influence of dissociated phoria and phoria adaptation is also a topic for future research (Kim et al., 2011). Furthermore, investigations studying patients with convergence or accommodation dysfunctions are warranted to determine how the preprogrammed component of accommodation stimulated by the Maddox components of vergence may differ compared to those with binocularly normal vision.

## Appendix A Appendix A: Clinical demographics for participants

**Table:**

Metric	Mean	Median	*SD*	Minimum	Maximum
Age (y)	21.7	21	3.6	18	34
CISS (points)	8.8	9	5.3	0	19
Near point of convergence, break (cm)	4.1	4	1.1	2	6
Near point of convergence, recovery (cm)	5.5	5.5	1.3	1	9
Near horizontal phoria (ΔD)	−2.1	−2	2.1	−6	3
Far horizontal phoria (ΔD)	−0.1	0	0.6	−3	0.5
Local stereopsis (arcsec)	31	30	13	20	70
Global stereopsis (arcsec)	250	250	0	250	250
Positive fusional vergence (ΔD)	26.6	25	8.8	16	45
Negative fusional vergence (ΔD)	13.7	14	2.6	8	20
Amplitude of accommodation, OD (D)	10.9	11	1.5	8	15
Amplitude of accommodation, OS (D)	10.8	10.6	1.4	8	15
OD sphere (D)	−0.7	0	1.7	−5.5	1
OS sphere (D)	−0.7	−0.3	1.7	−5.3	1.3

For phoria, exo deviation is denoted as negative and eso deviation is denoted as positive.

## Appendix B. Appendix B: Focusing and defocusing average movement descriptives with 95% confidence intervals

**Table:**

				95% confidence interval				
	Cue	*N*	Mean	Lower	Upper	*SD*	Min	Max
BNV focusing movement descriptives								
Peak velocity (D/s)	BDP	20	6.1	5.31	6.89	1.68	2.89	8.93
	BD(-p)	18	6.13	5.33	6.94	1.62	3.61	10.09
	BP(-d)	25	3.6	3.04	4.17	1.36	1.5	6.71
	DP(-b)	27	4.11	3.13	5.09	2.47	0.79	9.37
	B(-dp)	24	4.03	3.29	4.76	1.74	1.12	6.81
	D(-bp)	31	4.69	3.75	5.63	2.56	0.9	9.52
	P(-bd)	29	1.58	1.25	1.91	0.86	0.33	3.9
Final amplitude (D)	BDP	21	1.86	1.64	2.09	0.49	1.17	2.75
	BD(-p)	20	1.84	1.64	2.03	0.41	1.27	2.57
	BP(-d)	24	1.3	1.18	1.43	0.29	0.88	1.82
	DP(-b)	27	1.11	0.93	1.3	0.46	0.51	2.1
	B(-dp)	24	1.34	1.18	1.5	0.38	0.7	2.01
	D(-bp)	31	1.06	0.9	1.23	0.45	0.45	1.93
	P(-bd)	27	0.39	0.34	0.45	0.14	0.15	0.66
BNV defocusing movement descriptives								
Peak velocity (D/s)	BDP	21	−5.56	−6.14	−4.98	1.28	−7.73	−3.45
	BD(-p)	19	−6.98	−7.85	−6.12	1.8	−11	−4.86
	BP(-d)	27	−3.26	−3.85	−2.67	1.49	−6.87	−1.38
	DP(-b)	29	−3.89	−4.64	−3.14	1.98	−8.61	−0.82
	B(-dp)	24	−3.45	−4.06	−2.84	1.44	−6.34	−1.05
	D(-bp)	30	−4.15	−4.99	−3.3	2.27	−9.87	−0.87
	P(-bd)	29	−1.44	−1.78	−1.11	0.88	−3.63	−0.22
Final amplitude (D)	BDP	22	−1.85	−2.05	−1.65	0.44	−2.66	−1.14
	BD(-p)	20	−1.92	−2.12	−1.71	0.44	−2.71	−1.26
	BP(-d)	25	−1.3	−1.43	−1.17	0.31	−1.85	−0.83
	DP(-b)	29	−1.14	−1.31	−0.97	0.45	−2.02	−0.41
	B(-dp)	24	−1.33	−1.47	−1.18	0.34	−1.95	−0.67
	D(-bp)	30	−0.97	−1.14	−0.81	0.45	−1.84	−0.3
	P(-bd)	29	−0.45	−0.52	−0.38	0.18	−0.87	−0.14

BNV, binocularly normal vision.
